# Electrophysiology-Based Assays to Detect Subtype-Selective Modulation of Human Nicotinic Acetylcholine Receptors

**DOI:** 10.1089/adt.2015.688

**Published:** 2016-08-01

**Authors:** Glenn E. Kirsch, Nikolai B. Fedorov, Yuri A. Kuryshev, Zhiqi Liu, Lucas C. Armstrong, Michael S. Orr

**Affiliations:** ^1^ Charles River Discovery, Cleveland, Ohio.; ^2^ Center for Tobacco Products, US FDA, Silver Spring, Maryland.

## Abstract

*The Family Smoking Prevention and Tobacco Control Act of 2009 (Public Law 111-31) gave the US Food and Drug Administration (FDA) the responsibility for regulating tobacco products. Nicotine is the primary addictive component of tobacco and its effects can be modulated by additional ingredients in manufactured products. Nicotine acts by mimicking the neurotransmitter acetylcholine on neuronal nicotinic acetylcholine receptors (nAChRs), which function as ion channels in cholinergic modulation of neurotransmission. Subtypes within the family of neuronal nAChRs are defined by their α- and β-subunit composition. The subtype-selective profiles of tobacco constituents are largely unknown, but could be essential for understanding the physiological effects of tobacco products. In this report, we report the development and validation of electrophysiology-based high-throughput screens (e-HTS)* *for human nicotinic subtypes, α3β4, α3β4α5, α4β2, and α7 stably expressed in Chinese Hamster Ovary cells. Assessment of agonist sensitivity and acute desensitization gave results comparable to those obtained by conventional manual patch clamp electrophysiology assays. The potency of reference antagonists for inhibition of the receptor channels and selectivity of positive allosteric modulators also were very similar between e-HTS and conventional manual patch voltage clamp data. Further validation was obtained in pilot screening of a library of FDA-approved drugs that identified α7 subtype-selective positive allosteric modulation by novel compounds. These assays provide new tools for profiling of nicotinic receptor selectivity.*

## Introduction

Tobacco use is recognized as a significant, but avoidable health hazard. The Family Smoking Prevention and Tobacco Control Act, commonly referred to as the Tobacco Control Act, signed into law in 2009, gave the US Food and Drug Administration (FDA) immediate responsibility for regulating the manufacture, distribution, and marketing of tobacco products, specifically, cigarettes, cigarette tobacco, roll-your-own tobacco, and smokeless tobacco products. New tobacco products would have to undergo rigorous premarket FDA review. In addition, claims of reduced harm would have to be evaluated by the FDA to confirm that the scientific evidence supports the claim.

The Tobacco Control Act also gave the FDA the ability to extend the regulations—the so-called deeming rule—to cover all other categories of products that meet the Tobacco Control Act definition of “tobacco product.” These products include electronic cigarettes (e-cigarettes), cigars, pipe tobacco, waterpipe (hookah) tobacco, and novel products like nicotine gels, tobacco sticks, pellets and strips that dissolve in the mouth, and other dissolvable products not currently under FDA's regulatory authority as smokeless tobacco products.

Nicotine is the primary addictive component of tobacco products and additional ingredients may modulate nicotine's action at the receptor level.^1^ Nicotine acts by mimicking the endogenous neurotransmitter acetylcholine that activates ionotropic cholinergic receptors to depolarize neuronal membranes. Neuronal nicotinic acetylcholine receptors (nAChRs) are pentameric ligand-gated, cation-selective channels expressed in the central and peripheral nervous systems. Receptor subtypes are defined by their α- and β-subunit composition, and functional properties (*e.g.*, agonist sensitivity, pharmacologic profile, Ca^2+^ permeability, and desensitization kinetics).^2^

nAChRs in presynaptic terminals modulate the release of other neurotransmitters, for example, glutamate, γ-aminobutyric acid, and dopamine (DA). Brain circuits identified through functional brain imaging in humans and laboratory animal studies have demonstrated nicotine-induced activation of the prefrontal cortex, thalamus, and visual system, indicative of activation of cortico-basal ganglia-thalamic brain circuits, and increased DA concentration in the ventral striatum/nucleus acumbens.^3^ Multiple nAChR subtypes are expressed in these circuits and different subunits have been associated with different aspects of nicotine addiction. Evidence from mouse knockout studies^4,5^ has implicated α4, α6, and β2 subunits in the rewarding aspects of nicotine, whereas α5 contributes to the aversive effects of nicotine, and α5, α7, and β4 contribute to nicotine withdrawal symptoms. Apart from nicotine, the subtype-selective profiles of other tobacco product constituents are largely unknown and would be of importance in understanding their addiction potential.

This study describes the development and validation of high-throughput electrophysiology-based assays in four recombinant cell lines, each expressing a different subtype of human nicotinic receptor: α3β4, α3β4α5, α4β2, and α7/RIC-3. Receptors were expressed by stably transfecting Chinese Hamster Ovary (CHO) parental cells. Assays for each subtype were optimized and pharmacologically validated with reference compounds in an automated electrophysiology system (IonWorks Barracuda^®^ [IWB]; Molecular Devices, LLC, Sunnyvale, CA).

## Materials and Methods

### Cell Lines

Stable cell lines constitutively expressing human nAChRs were constructed as described previously.^6^ Ion channel cDNAs were cloned by reverse transcription–polymerase chain reaction from commercially available human RNAs (Clontech, Mountainview, CA). Full-length cDNAs were assembled in mammalian expression vectors for constitutive expression. Mammalian expression vectors with different antibiotic selectable markers (geneticin, hygromycin, and zeocin) were used for coexpression of multiple subunits. Before transfection, all cDNAs were sequenced in their entirety and the translated sequences were found to be identical to protein sequences in GenBank as follows: *CHRNA3* (α3), NM_000743.2; *CHRNA4* (α4), NM_000744.2; *CHRNA5* (α5), NM_000745.2; *CHRNA7* (α7), NM_000746.3; *CHRNB2* (β2), NM_000748.1; *CHRNB4* (β4), NM_000750.3; and *RIC3* (RIC-3), NM_024557.3.

Transfection-ready cDNAs were linearized in nonessential regions of the plasmid to facilitate incorporation into the host cell genomic DNA. Host cells (CHO) were maintained in Ham's F-12 media supplemented with 10% fetal bovine serum, 100 U/mL penicillin G sodium, and 100 μg/mL streptomycin sulfate, passaged twice weekly by washing with Hank's Balanced Salt Solution (HBSS) lacking calcium and magnesium, and detached with Accutase™ (Innovative Cell Technologies, San Diego, CA). CHO cells were transfected with the linearized cDNA constructs by nucleofection (Amaxa, Cologne, Germany) using the kit for CHO cells following manufacturer's instructions. Transfected cells were incubated with the appropriate selection antibiotics for 2 weeks to select stably expressing cells. Selection antibiotics were included as follows: α3β4 and α7/RIC-3, 250 μg/mL geneticin (G418) and 400 μg/mL zeocin; α3β4α5, 250 μg/mL geneticin (G418), 400 μg/mL zeocin, and 8 μg/mL puromycin; and α4β2, 400 μg/mL hygromycin and 8 μg/mL puromycin. Individual subclones were then selected, expanded, and assayed for functional channel expression on IWB.

To prepare cells for electrophysiological experiments, CHO cells were routinely maintained in growth media containing the appropriate selection antibiotics, as described above. At 2–4 days before the experiments, the cells were passed in a medium lacking selection antibiotics. Cell density was ∼50%–70% confluent at the time of harvest; two 150 mm plates (∼1.2 × 10^7^ cells) were used per population patch clamp (PPC) experiment.

Cells were harvested by washing twice with 15–20 mL of HBSS lacking calcium and magnesium and treatment with 5 mL of Accutase solution for 20 min. Cells were resuspended in a 50-mL conical tube with the addition of 10 mL of HBSS and triturated with a serological pipette to resuspend the cells and break up cell clusters. Cells were pelleted at 500 *g* for 2.5 min, the supernatant was removed, and the cell pellet was resuspended in 10 mL of HBSS. The cell suspension was centrifuged again at 500 *g* for 2.5 min and the supernatant removed. Finally, the cell pellet was resuspended in 5 mL of HEPES-buffered physiological saline (HBPS).

### Solutions and Electrophysiological Procedures

Chemicals used in solution preparation were purchased from Sigma-Aldrich (St. Louis, MO) and were of ACS reagent grade purity or higher. Stock solutions of test articles were prepared in dimethyl sulfoxide (DMSO) and stored frozen. Each test article formulation was sonicated (Model 2510/5510; Branson Ultrasonics, Danbury, CT) at ambient room temperature for 20 min to facilitate dissolution. Test article concentrations were prepared fresh daily by diluting stock solutions into the extracellular solution (HBPS buffer). The solution composition was 137 mM NaCl, 4 mM KCl, 1.8 mM CaCl_2_, 1 mM MgCl_2_, 10 mM HEPES, and 10 mM glucose, pH adjusted to 7.4 with NaOH. The osmolarity was adjusted to 295 ± 5 mOsm. All test and control solutions contained 0.3% DMSO and 0.05% F-127. The test article formulations were prepared in 384-well compound plates using an automated liquid handling system (Cyclone, Caliper).

The internal HEPES-buffered solution consisted of 90 mM CsF, 50 mM CsCl, 2 mM MgCl_2_, 5 mM EGTA, and 10 mM HEPES, pH 7.2 adjusted with CsOH. The osmolarity was adjusted to 275 ± 5 mOsm. Stock solution of Amphotericin B was prepared in DMSO (30 mg/mL) and added to the solution at the final concentration of 33.3 μg/mL. The extracellular buffer was loaded into the PPC plate wells (11 μL/well) and the cell suspension was added into the wells (9 μL/well). After establishment of a whole-cell configuration (10 min perforation), membrane currents were recorded by on-board patch clamp amplifiers in the IWB. The data acquisition frequency was 5 kHz. Inward current peak amplitudes and charge movement (area-under-the-curve [AUC] during the 5-s interval starting at solution addition, unless otherwise specified) were measured. Under these conditions, each assay was completed in 45 min, and up to 10 experiments per instrument could be conducted during an 8-h day.

Ionic currents were elicited with the application of 20 μL agonist (10 μL/s). Antagonists were preincubated for 5 min before application of (−)-nicotine at a concentration to produce 90% (EC_90_) of the maximum response (EC_Max_). To evaluate effects of positive modulators, currents were elicited with (−)-nicotine at a concentration sufficient to produce 20% (EC_20_) of the EC_Max_. Recordings were started 2 s before the addition with the total recording duration of 17 s. The holding potential was −70 mV. A summary of the assay protocol is presented in *Table 1*.

**Table 1.** Assay Protocol

Step	Parameter	Value	Description
1	Prepare compound plate	50 μL/well	2 × concentrated test compound and controls
2	Load internal solution		Includes perforant, Amphotericin B at 33.3 μg/mL
3	Load extracellular solution into assay plate	11 μL/well	
4	Dispense cells	9 μL/well	2 × 10^7^ cells/plate
5	Establish whole-cell configuration	−70 mV	10 min patch perforation with amphotericin
6	Test compound addition	20 μL/well	Record ionic current for 5 s at 5 kHz sampling frequency
7	Nicotine stimulus addition	20 μL/well	Replace 20 μL to reach 40 μL/well final volume, record ionic current

**Step Notes**

1. 384-well compound plates loaded with test and control compounds from DMSO stock diluted in extracellular solution (final DMSO concentration, 0.3% v/v).

2. Composition of internal solution presented in text.

3. Composition of extracellular solution presented in text.

4. Cells suspended in extracellular solution at 4 × 10^6^ cells/mL.

5. Perforated patch whole-cell configuration with amphotericin B stock prepared fresh in DMSO at 30 mg/mL.

6. Recording initiated ∼1 s before addition. Exposure to test or control compounds, 5 min.

7. 5 s recording upon ligand stimulus addition.

DMSO, dimethyl sulfoxide.

### FDA-Approved Drug Library

A library of 786 FDA-approved drugs was purchased from Enzo Life Sciences (Screen-Well™ compound library, BML-2843-0100; Farmingdale, NY). Compounds were received as 100 μL samples dissolved mainly in DMSO (except for one compound in water) at 10 mM. Daughter plates were prepared in a 384-well format and compounds were initially screened at a final concentration of 60 μM. Potency of actives was measured at concentrations up to 300 μM. Screening and potency confirmation experiments were conducted in an agonist/positive modulator mode by preincubation with test compound for 5 min followed by challenge with test compound plus ligand (nicotine) at ∼EC_20_ concentration.

### Data Analysis

Data acquisition and analyses were performed using the IWB system software (version 2.0.0.335). Data were corrected for leak current. Off-line data analysis was performed in Microsoft^®^ Excel^®^ (2013; ver. 15.0.4779.1001; Redmond, WA).

### Activation Calculation

nAChR activation was calculated as follows:

\begin{align*}
\% \ { \rm{ Activation}} = \left( {{{ \rm{I}}_{{ \rm{Agon}}}} / {{ \rm{I}}_{{ \rm{Max}}}}} \right) \times 100 \% ,
\end{align*}

where I_Agon_ was the agonist-elicited current signal and I_Max_ was the mean current signal elicited with 300 μM (−)-nicotine.

Concentration–response data were fitted to an equation of the following form:

\begin{align*}
\% \ { \rm{ Activation }} = \% \ { \rm{ VC}} + \{  ( \% \ { \rm{ Max}} - \% \ { \rm{ VC}} ) / \left[ {1 } + ( \right[ { \rm{Test}} \left] { / { \rm{E}}{{ \rm{C}}_{{ \rm{5}}0}}{ ) ^{ \rm{N}}}} \right] \}  ,
\end{align*}

where [Test] was the concentration of agonist, EC_50_ was the concentration of agonist producing half-maximal activation, N is the Hill coefficient, % VC was the percentage of the current signal at addition (the mean current at the DMSO vehicle control addition), % Max is the percentage of the current activated by the highest dose of (−)-nicotine, and % Activation is the percentage of the current signal elicited at each concentration of agonist. Nonlinear least squares fits were solved with the XLfit add-in for Excel 2003 (Microsoft). EC_20_ and EC_90_ values were calculated from the fitted curves.

### Inhibition Calculation

Inhibitory effects were calculated as follows:

\begin{align*}
\% \ { \rm{ Block }} = ( 1 - {{ \rm{I}}_{{ \rm{TA}}}} / {{ \rm{I}}_{{ \rm{Control}}}} ) \times 100 \% ,
\end{align*}

where I_TA_ was the (−)-nicotine-elicited current in presence of a test article and I_Control_ was the mean (−)-nicotine EC_90_-elicited current.

Antagonist concentration–response data were fit to an equation of the following form:

\begin{align*}
\% \ { \rm{Change }} = \% \ { \rm{Min }} + \{  ( \% \ { \rm{ Max}} - \% \ { \rm{ Min}} ) / \left[ 1 + ( \right[ { \rm{Test}} \left] { / { \rm{I}}{{ \rm{C}}_{50}}{ ) ^{ \rm{N}}}} \right] \}  ,
\end{align*}

where [Test] was the concentration of a test article, IC_50_ was the concentration of the test article producing half-maximal inhibition, N is the Hill coefficient, % Min was the mean current elicited with (−)-nicotine EC_90_ plus vehicle control, % Min was the current measured at the DMSO vehicle addition, and % Block was the percentage of the current inhibited at each concentration of a test compound.

### Potentiation Calculation

Potentiating effects of positive allosteric modulators (PAMs) on the channels were calculated as follows:

\begin{align*}
\% \ { \rm{Activation }} = \left( {{{ \rm{I}}_{{ \rm{PAM}}}} / {{ \rm{I}}_{{ \rm{Max}}}}} \right) \times 100 \% ,
\end{align*}

where I_PAM_ was the (−)-nicotine ∼EC_20_-elicited current in presence of the PAM and I_Max_ was the mean current elicited at the highest (−)-nicotine.

PAM concentration–response data were fitted to an equation of the following form:

\begin{align*} \% \ { \rm{Activation }} & = \% \ { \rm{Nicotine }} \ { \rm{E}}{{ \rm{C}}_{{ \rm{2}}0}} \\ & + \{  ( \% \ { \rm{Max}} - \% \ { \rm{Nicotine \ E}}{{ \rm{C}}_{{ \rm{2}}0}} ) / \left[ 1 + ( \right[ { \rm{Test}} \left] { / { \rm{E}}{{ \rm{C}}_ {50}}{ ) ^{ \rm{N}}}} \right] \}  ,\end{align*}

where [Test] was the concentration of PAM, EC_50_ was the concentration of PAM producing half-maximal activation, N is the Hill coefficient, % nicotine EC_20_ was the percentage of current elicited with nicotine at the EC_20_ concentration, % Max is the percentage of current activated with the highest dose of nicotine, and % Activation is the percentage of current elicited with nicotine EC_20_ at each PAM concentration.

### Acceptance Criteria

Individual well data were filtered according to electrical criteria and the experiments were accepted based on plate-level criteria (*Table 2*).

**Table 2.** Acceptance Criteria

Parameter	Acceptance criterion (PPC well level)
R_SEAL_ (Baseline)^a^	>100 MΩ
Current amplitude (Baseline)	>0.2 nA
R_SEAL_ stability (between first and second additions)	<50% decrease

	Acceptance criterion (PPC plate level)
Z′ factor	≥0.5
Success rate (% valid wells)	>90% accepted wells per PPC plate
EC/IC_50_ for reference compounds	≤0.5 log from historical mean

^a^Typical R_seal_ values ranged between 200 and 1,000 MΩ.

PPC, population patch clamp.

Z′ factor in each PAM experiment was calculated as follows:

\begin{align*}
{ \rm{Z  ^\prime }} = { \rm{ 1}} - ( { \rm{3}} \times { \rm{S}}{{ \rm{D}}_{{ \rm{Min}}}} + { \rm{ 3}} \times { \rm{S}}{{ \rm{D}}_{{ \rm{Max}}}} ) / { \rm{ABS }} ( { \rm{Mea}}{{ \rm{n}}_{{ \rm{Min}}}} - { \rm{Mea}}{{ \rm{n}}_{{ \rm{Max}}}} ),
\end{align*}

where Mean_Min_ and SD_Min_, respectively, were the mean and standard deviation values for the minimum signal (Min). Mean_Max_ and SD_Max_, respectively, were the mean and standard deviation values for the Maximum signal (Max).

For α3β4, α3β4α5, and α4β2 receptors, Min was measured at stimulation with 3 μM (−)-nicotine and Max was measured at stimulation with 300 μM (−)-nicotine. For α7 receptors, Min was measured at stimulation with 20 μM (−)-nicotine and Max was measured at stimulation with 1 μM PNU120596 plus 20 μM (−)-nicotine.

## Results

### Functional Confirmation of α3β4α5 Expression in CHO Cells

**Fig. 1.**
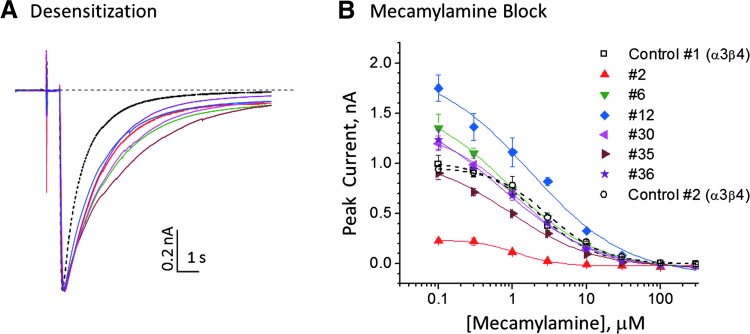
Functional confirmation of α3β4α5 expression. Cryopreserved cells were thawed, washed, and dispensed to the assay plate without culturing. Cells from the parental cell line (α3β4) and α3β4α5 clones #2, #6, #12, #30, #35, and #36 were tested in the antagonist mode [100 μM (−)-nicotine stimulation, population patch mode]. **(A)** Current traces normalized to the peak response. Traces were analyzed by fitting to single-exponential decay functions. Time constants (mean ± SEM) of 1.0 ± 0.04 s (*n* = 9) and 1.8 ± 0.07 s (*n* = 6), respectively, were obtained in α3β4 and α3β4α5 receptors. The slower decay of α3β4α5 currents (*solid colored traces*) in the presence of ligand compared to the parental α3β4 cells (*broken black trace*) is a characteristic of α5 expression.^8^ **(B)** Mecamylamine concentration–response relationships were measured at concentrations of 0.1–300 μM. α5 transfection increased the sensitivity to mecamylamine ∼2-fold compared to the parental cell line. Data were fitted to one-site model. IC_50_ values (mean ± SEM) 2.7 ± 0.5 μM (*n* = 2) and 1.1 ± 0.4 μM (*n* = 6), respectively, were obtained in α3β4 and α3β4α5 receptors. SEM, standard error of the mean.

The α5 subunit, which lacks critical residues necessary to form a ligand-binding site, has been considered to be an auxiliary protein that modifies functional receptor characteristics. The α3β4α5 subtype is distinguished from α3β4 by increased sensitivity to the noncompetitive antagonist, mecamylamine,^7^ and slower rate of desensitization.^8^ A stably transfected α3β4-CHO cell line (CT6021; ChanTest Corporation, Cleveland, OH) was subsequently transfected with an expression vector encoding the α5 subunit. We compared nicotine-induced response of the parental α3β4-CHO cell line to that of several independent α3β4α5-CHO clones in the same experiment. *Figure 1A* shows that when scaled to peak response, the current waveform for all six α3β4α5-CHO clones decayed more slowly compared with the α3β4-CHO parental cells. As shown in *Figure 1B*, mecamylamine potency was increased twofold in the α3β4α5-CHO clones compared to the α3β4-CHO control. These results together with detection of α5 protein expression by Western blot (data not shown) confirmed the expression of α3β4α5 receptor subtypes in our cell line.

**Fig. 2.**
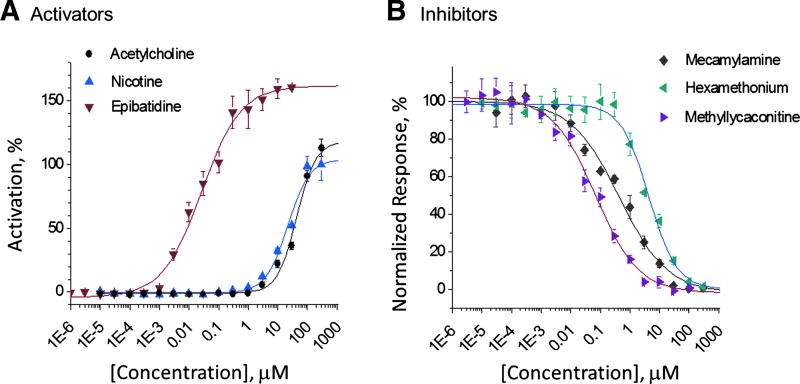
α3β4α5 agonist **(A)** and antagonist **(B)** evaluations. Concentration–response curves (16-point CRC) were obtained for reference compounds in two independent experiments on different days. Consistent EC/IC_50_ values were obtained both days (day 1 shown). Data points represent mean ± SEM (three to four replicate wells/concentration). In the antagonist experiments, the cells were preincubated for 5 min with test compound at the indicated concentrations. Currents were elicited with application of 100 μM (−)-nicotine. Agonist data were normalized to the (−)-nicotine maximum current amplitude. Data were fitted to one-site models.

We characterized the potencies of reference agonists and antagonists as shown in *Figure 2*. Application of agonists (acetylcholine, nicotine, and epibatidine, *Fig. 2A*) elicited concentration-dependent activation of inward ionic currents. The percent activation was calculated from the peak current amplitude normalized against the maximum amplitude at 300 μM nicotine. The rank order of potency based on EC_50_ values was epibatidine > nicotine > acetylcholine. It is noteworthy that the maximum response to epibatidine exceeded that of either nicotine or acetylcholine, and the EC_50_ values of the latter compounds were right-shifted compared to that of epibatidine, suggesting that epibatidine has both higher affinity and greater efficacy.

Antagonist validation (*Fig. 2B*) included the noncompetitive inhibitors mecamylamine and hexamethonium, and the competitive antagonist, methyllycaconitine (MLA). The rank order of potency based on IC_50_ values was methyllycaconitine > mecamylamine > hexamethonium.

### Validation of Agonist, Antagonist, and Modulator Assays in α4β2 and α7 Subtypes

**Fig. 3.**
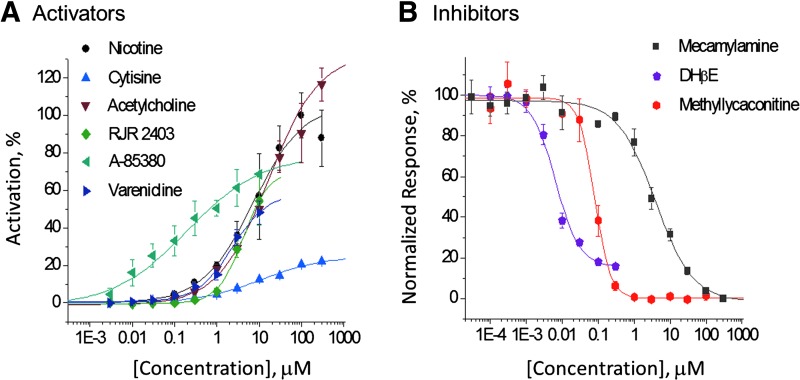
α4β2 agonist **(A)** and antagonist **(B)** assay evaluations. Concentration–response curves were obtained for six reference agonists and three inhibitors in two independent experiments on different days (day 1 shown). Data points represent mean ± SEM (two to four replicate wells/concentration). In the antagonist experiments, the cells were preincubated for 5 min with test compound at the indicated concentrations. Currents were elicited with application of 100 μM (−)-nicotine. Agonist data were normalized to the (−)-nicotine maximum current amplitude. Data were fitted to one-site models.

The α4β2 subtype is a validated therapeutic target in treatment of nicotine dependence and is a potential target for treatment of addiction to other psychoactive drugs as well, for example, alcohol, cocaine, and amphetamine.^9^ Tobacco addiction, for instance, has been treated with the α4β2 partial agonist, varenicline (Chantix^®^; Pfizer, New York, NY), and α4β2 antagonists have been tested clinically to augment serotonin-selective reuptake inhibitors in treatment of depression.^10^ Therefore, we optimized assays in agonist, antagonist, and PAM modes. In the agonist mode (*Fig. 3A*) cytisine and A-85380 showed lower efficacy than nicotine and acetylcholine. The rank order of potency (*Table 3*) was as follows: A-85380 > varenicline > RJR2403 > nicotine > cytisine.

**Table 3.** α4β2 Agonist Potency

	EC_50_, μM			
Reference agonist	Day 1	Day 2	Average	Reference
A-85380	0.197	0.291	0.24	0.4^a^
Acetylcholine	16.89	27.80	22.3	1.3 (HS); 75.3 (LS)^b^
Cytisine	11.65	10.34	11.0	11.6^c^
Nicotine	7.12	9.77	8.4	2.5^a^
RJR 2403	4.21	3.47	3.8	10^a^
Varenicline	2.33	1.48	1.9	4^a^

^a^Ref.^46^: FLIPR Ca-flux.

^b^Ref.^22^: IWB, HS.

^c^Ref.^47^: Manual patch clamp.

FLIPR, Fluorometric Imaging Plate Reader; HS, high sensitivity; IWB, IonWorks Barracuda; LS, low sensitivity.

The EC_50_ values obtained in these experiments are consistent with activation of both the high- and low-sensitivity components reported previously in cRNA-injected *Xenopus laevis* oocytes.^11^ Direct evidence for multiple components in our α4β2-CHO cell line is presented below.

**Fig. 4.**
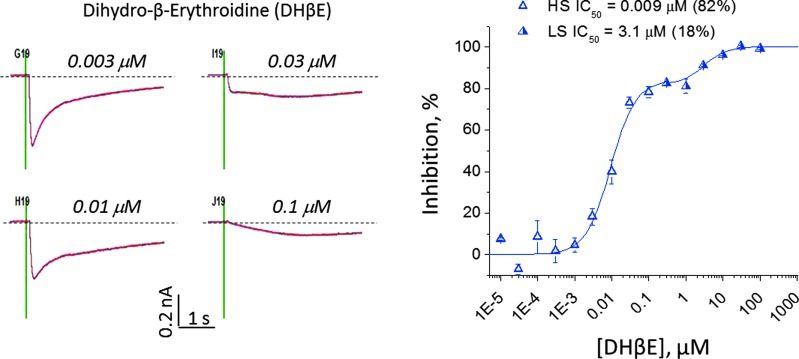
Evidence for two levels of DHβE inhibition of α4β2. The concentration–response relationship showed an apparent inflection point between 1 and 3 μM, suggestive of high-sensitivity and low-sensitivity components. **(A)** Representative examples of residual currents (low sensitivity) at 0.1 μM DHβE and a mixture of high and low sensitivity at concentrations of 0.003–0.03 μM. **(B)** Full concentration–response curve fitted to a two-site model. Data points represent mean ± SEM (three to four replicate wells/concentration). DHβE, dihydro-β-erythroidine.

Reference compounds dihydro-β-erythroidine (DHβE), MLA, and mecamylamine were tested in the antagonist mode (5-min preincubation followed by stimulation with 100 μM nicotine, *Fig. 3B*). We found that DHβE was the most potent and mecamylamine the least potent inhibitor. It is noteworthy that the fitted DHβE curve did not reach 100% inhibition at a concentration 50-fold greater than the IC_50_ value (0.006 μM). *Figure 4* extends the analysis of the concentration–response relationship up to 100 μM. We observed a notable inflection point between 1 and 3 μM, consistent with the presence of both high-sensitivity (IC_50_ = 0.009 μM) and low-sensitivity (IC_50_ = 3.1 μM) components. These components may represent heterogeneity in subunit composition as reported previously.^11^ Fitting the concentration–response relationship to a two-site model indicated that the high- and low-sensitivity components, respectively, represent 82% and 18% of the channel population.

**Fig. 5.**
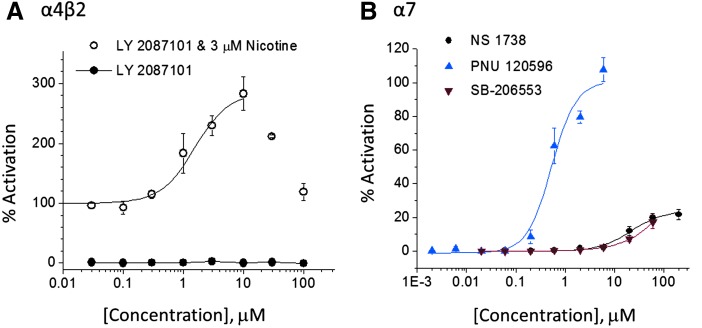
Positive modulation of α4β2 and α7 receptors. **(A)** α4β2-CHO cells were preincubated with LY2087101 alone (addition #1) at the indicated concentrations and then stimulated with 3 μM (−)-nicotine (addition #2). Percent activation was calculated by normalizing against vehicle controls activated by 3 μM (−)-nicotine alone (100% activation). Concentration–response curves (eight point) were obtained in two independent experiments on different days (day 1 illustrated). Data points represent mean ± SEM (two to four replicate wells/concentration). *Filled symbols* represent addition #1 (LY2087101 in the absence of ligand), *open symbols* show peak currents recorded during addition #2. Data points were fitted with EC_50_ = 1.1 μM. **(B)** α7/RIC-3-CHO cells were preincubated with PNU120596, NS1738, or SB-206553 alone at the indicated concentrations and then stimulated with 20 μM (−)-nicotine. Percent potentiation was normalized against maximal signal [20 μM (−)-nicotine with 10 μM PNU120596]. Data were fitted with EC_50_ values, 21.7, and 0.6 μM, respectively, for NS 1738 and PNU 120596. The EC_50_ value for SB-206553 was greater than 50 μM. CHO, Chinese hamster ovary.

We verified the sensitivity of the IWB assay to a PAM (α4β2-selective reference compound LY2087101, *Fig. 5A*). Preincubation with LY2087101 at concentrations up to 100 μM (filled symbols) had no effect in the absence of the agonist, nicotine. In the second addition, coapplication of ascending concentrations of LY2087101 + 3 μM nicotine gave a concentration-dependent potentiation of peak current amplitude consistent with positive allosteric modulation. The EC_50_ value calculated at LY2087101 concentrations of 0.03–10 μM averaged 1.19 μM in independent experiments conducted on different days, consistent with published values.^12^ At 10 μM, we observed maximum potentiation of 280%. At concentrations above 10 μM, the potentiation decreased such that at 100 μM LY2087101, the response returned to the same level as 3 μM nicotine alone. A similar observation has been reported previously.^12^

The α7 subtype has high permeability to Ca^2+^ ions, relative to other nAChRs, and is a potential target for treatment of Parkinson's disease,^13^ inflammatory bowel disease,^14^ and cognition deficits in schizophrenia^15^ and Alzheimer's disease.^16^ We constructed a cell line coexpressing the α7 subunit with RIC-3, a chaperone that increases trafficking of several nAChR subtypes to the cell surface.^17^ We conducted positive modulator experiments in α7/RIC-3-CHO cells in the same manner as shown for the α4β2 subtype and obtained α7 EC_50_ values presented in *Table 4* and *Figure 5B*.

**Table 4.** α7 Positive Allosteric Modulator EC_50_ Values

	EC_50_, μM			
PAM	Day 1	Day 2	Average	Reference
NS 1738	21.7	38.6	30.2	12.5^a^
PNU 120596	0.527	0.589	0.558	0.216^b^
SB-206553	>5 μM	>5 μM	>5 μM	45^c^

^a^Ref.^19^: QPatch ionic current.

^b^Ref.^48^: FLIPR Ca^2+^ flux.

^c^Ref.^49^: FLIPR Ca^2+^ flux.

PAM, positive allosteric modulator.

### Identification of Subtype-Selective Modulators

**Fig. 6.**
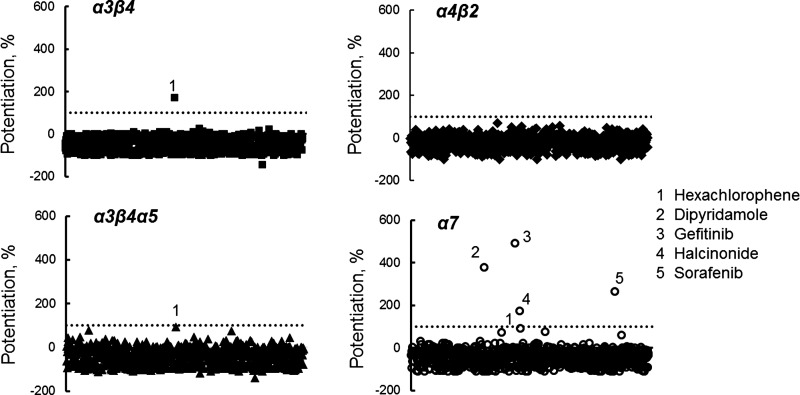
Screen of Enzo Library for allosteric modulators. The library of 786 FDA-approved drugs was screened at 60 μM in the agonist/PAM mode. Drugs were preincubated for 5 min to identify agonists and then stimulated with (−)-nicotine at ∼EC_20_ concentration. The *dotted line* represents 100% increase in signal relative to vehicle controls. Data points that exceeded this threshold were identified as “hits.” FDA, US Food and Drug Administration; PAM, positive allosteric modulator.

We tested the feasibility of using IWB as a tool for identifying subtype-selective modulators by conducting a pilot screen of the Enzo Screen–Well library of 786 FDA-registered drugs against all four nicotinic subtypes: α3β4, α3β4α5, α4β2, and α7 in agonist, antagonist, and PAM modes. *Figure 6* presents the results of single-point screening of the library in the agonist/PAM mode [preincubation with test compound alone at 60 μM for 5 min followed by challenge with (−)-nicotine at ∼EC_20_ concentration]. We defined PAM actives as drugs that increased normalized peak current by at least 100% relative to the average response in the vehicle control wells after (−)-nicotine challenge. Compounds that acted as agonists during preincubation in the absence of (−)-nicotine stimulation or as antagonists in the presence of (−)-nicotine were excluded.

**Fig. 7.**
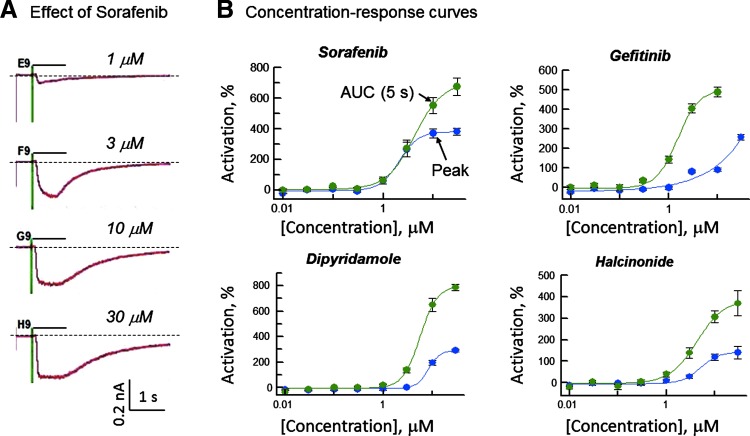
α7 PAM confirmation concentration–response relationships. **(A)** Example traces illustrating concentration-dependent effects of sorafenib in PAM mode. Currents were elicited by application of 20 μM (−)-nicotine in presence of sorafenib. **(B)** Concentration–response curves obtained from integrated current records (5 s, AUC). EC_50_ values (AUC measurement) were as follows: Sorafenib (4.2 μM), Gefitinib (1.5 μM), Dipyridamole (5.5 μM), and Halcinonide (4.3 μM). Data presented as mean ± SEM (*n* = 3–4 wells/concentration). AUC, area under the curve.

We found that hexachlorophene (data point #1) acted as a weak, nonselective PAM in three of the four nAChR subtypes. In addition, we identified four subtype-selective positive modulators in the α7 subtype (*Fig. 6*, data points #2–#5). The α7 PAM hits were confirmed in a separate potency experiment as shown in *Figure 7*. Two of the confirmed hits were antineoplastic agents (gefitinib and sorafenib). A phosphodiesterase inhibitor (dipyridamole) and a corticosteroid (halcinonide) also were confirmed. We noted that in all instances, the positive modulation was concentration dependent with thresholds in the range 0.1–1 μM. Also, as exemplified by sorafenib in *Figure 7A*, the confirmed hits appeared to modify channel gating by slowing the time course of densensitization (a type II PAM response, similar to that of PNU120596) rather than simply scaling the current waveform. Thus, analysis of integrated charge movement (AUC *Fig. 7B*) resulted in an apparently higher level of potentiation than measurement of peak response.

This single-point PAM screen also provides a representative snapshot of assay performance as presented in *Table 5*. The screen, conducted against the four receptor targets, required three assay plates/target; all plates passed acceptance criteria based on Z′ values (mean values ranged 0.547–0.764) and success rate (% of accepted wells in each plate >95%).

**Table 5.** Enzo Library Positive Allosteric Modulator Screen

	Z′ value			Success rate, % accepted wells/plate	
Receptor subtype	Mean	SD	*N* (plates)	Mean	SD
α3β4	0.656	0.054	3	97.6	0.8
α4β3	0.547	0.020	3	99.7	0.2
α3β4α5	0.585	0.050	3	98.9	0.6
α7	0.764	0.111	3	98.4	0.8

SD, standard deviation.

## Discussion

The Tobacco Control Act gave the US FDA authority to regulate the manufacturing, marketing, and distribution of tobacco products, including developing science-based regulations such as product standards to change the amount of ingredients, including additives that can affect the sensory and pharmacological effects of the products by interaction with nAChRs. We selected the IWB automated patch-clamp system for its ability to screen thousands of compounds per day and profile multiple receptor subtypes expressed in recombinant cell lines. Assays were validated against reference compounds with known nicotinic interaction in selectivity profiling mode and in a small library of drugs that was evaluated in single-point screening mode. Actives selective for positive allosteric modulation of α7 receptors were confirmed in concentration–response mode.

Rapid screening of nAChRs in pharmaceutical research traditionally has been performed using high-throughput radioligand binding assays in membrane fractions or cell-based functional assays using fluorometric detection of changes in intracellular Ca^2+^ or membrane potential. Typically, follow-up confirmation of actives and selectivity profiling assays are performed by low-throughput electrophysiological recording in manual patch clamp of mammalian cells or intracellular microelectrode voltage clamp in *Xenopus* oocytes. More recently, automated patch-clamp technology that uses multiwell planar electrodes has become an alternative approach to screening and profiling. Several published studies have shown the utility of this technology in nicotinic receptor research. Characterization of the α7 nAChR subtype has been reported by several groups that used PatchXpress (16 well; Molecular Devices),^18^ QPatch (16 and 48 well; Sophion, Ballerup, Denmark),^19,20^ or SynchroPatch (96 well; Nanion, Munich, Germany)^21^ systems. Similar to our results, α7 assays performed on these platforms were shown to be effective in identifying PAMs and to require coapplication of the PAM for accurate measurement of agonist and antagonist potency owing to the rapid desensitization characteristic of α7 receptors. In contrast to these other platforms, IWB has the advantages of a 384-well format offering relatively high throughput (up to 3,500 compounds per 8-h day in single-point screen or 100 compounds per day in 8-point potency profiling) and population patch clamp configuration at 64 holes per well providing improved uniformity over single-hole planar electrodes. A disadvantage of the IWB platform, however, is its inability to conduct washout experiments to measure reversibility of drug effects and inability to run unattended. It is anticipated that newer 384-well platforms (*e.g.*, Sophion Qube and Nanion Synchropatch) will address these issues.

Evaluation of α3β4 and α4β2 pharmacology also has been reported previously in automated patch clamp (PatchXpress and IWB) with parallel experiments in manual patch-clamp assays.^22^ Their results show good agreement between the three platforms and were consistent with our results. For instance, we obtained a mecamylamine IC_50_ value of 2.7 μM (*Fig. 1*) in IWB versus their value of 1.2 μM in IWB and 3.3 μM in manual patch clamp. It should be noted the same cell line (α3β4-CHO; ChanTest Corporation) was used by both laboratories. The consistency of the results between laboratories and platforms argues for the utility of IWB assays in nicotinic screening and profiling. In the α4β2 experiments, both studies found evidence of a mixture of high- and low-sensitivity ligand-binding sites, suggestive of a mixed population of α4β2 receptors with different subunit stoichiometries.^11^ In our case, the heterogeneity was observed in the biphasic concentration–response relationship of DHβE, a competitive inhibitor, whereas Graef *et al.* observed two-site behavior in the acetylcholine concentration–response relationship. This difference may be related to differences between the background parental cell line—CHO in our case and human epithelial SH-EP1 in theirs.

The α3β4 and α3β4α5 nAChR subtypes, highly expressed in autonomic ganglia, participate in regulation of heart rate and blood pressure, and may be responsible for cardiovascular effects of nicotine consumption.^23^ In addition, α3β4α5 receptors, localized in the habenulo-peduncular tract, are thought to contribute to nicotine-related effects, including reinforcement, aversion, and withdrawal.^24^ The α5 subunit, which lacks critical residues necessary to form a ligand-binding site, is considered to be an auxiliary protein that modifies functional receptor characteristics. Nicotinic receptors that contain the α5 subunit are of particular interest because of their potential involvement in nicotine addiction,^25^ and other health conditions.^26,27^ We compared the gating and pharmacological characteristics of α3β4 and α3β4α5 subtypes in automated patch clamp to show for the first time that a high-throughput patch-clamp platform is capable of detecting the effects of the auxiliary α5 subunit on α3β4 desensitization and inhibitor potency previously obtained by microelectrode voltage clamp with *Xenopus* oocytes and manual patch clamp with mammalian cells, each expressing α3β4α5.^7,8^ Experimental conditions were optimized for sensitivity and reproducibility by recording in the cell population mode (PPC) and adjusting the positive control agonist and DMSO concentrations. At 0.3% DMSO, we observed Z′ values (a statistical measure of assay quality that includes both variability and signal dynamic range) that exceeded 0.5 indicative of a robust assay.

The α4β2 subtype is the most abundant nicotinic receptor in the brain and is known to be expressed in the ventral tegmental region associated with addictive nicotine-induced DA release.^28^ Its importance in nicotine addiction has been demonstrated by observations that nicotine self-administration is lost in β2 knockout mouse models,^29^ chronic nicotine administration upregulates α4β2 receptor expression and nicotine sensitivity in binding studies,^30^ and notably, α4β2 is a primary target of varenicline, an approved drug for treatment of smoking cessation.^31,32^ Our results show that the IWB platform is capable of distinguishing both high- and low-sensitivity components of drug binding at the orthosteric agonist site by demonstrating the biphasic concentration–response relationship to the competitive antagonist DHβE. The presence of a mixture of both high- and low-sensitive components has been reported in previous *Xenopus* oocyte experiments and has been shown to arise from differences in subunit stoichiometry.^11^

It is notable that prolonged nicotine exposure *in vivo* in both humans^33^ and animal models^34^ has been shown to upregulate α4β2 receptor expression. A similar phenomenon has been shown to occur in heterologous expression in mammalian cells where subacute nicotine pretreatment increases the number of functional α4β2 receptors and shifts the stoichiometry to produce more high-sensitivity receptors.^35,36^ Therefore, the proportion of high (82%)- and low-sensitivity (18%) receptors in our recombinant cell line may be relevant to the native condition in chronic nicotine exposure.

To test whether our assays were capable of profiling nicotinic receptors for subtype-selective modulatory effects, we screened a library of 786 drugs (Screen-Well FDA Approved Drug Library; Enzo Life Sciences) against nAChRs in the PAM mode. We identified four α7-selective compounds that to our knowledge have not been shown previously to act as PAMs in nicotinic receptors (*Figs. 6* and *7*): dipyridamole, gefitinib, halcinonide, and sorafenib. Gefitinib and sorafenib are tyrosine kinase inhibitors used in cancer treatment. They are known to have diverse secondary pharmacology. Gefitinib, for instance, inhibits (K_i_ values 1–4 μM) DA D3 receptors, muscarinic cholinergic receptors M1 and M4, α1d-adrenergic receptors, tachykinin NK2 receptors, and serotonin 5-HT2b receptors, and human *Ether-à-go-go* related gene (hERG) potassium channels.^37^ Similarly, sorafenib interacts with multiple cell surface targets,^38,39^ including several kinases (*e.g.*, FLT-3, VEGFR-2, and PDGFR-β) and ion channels (hERG potassium channel and Cav1.2 calcium channel).

Dipyridamole, an adjunct to anticoagulant therapy, also shows a broad pharmacological activity, including inhibition of adenosine reuptake^40^ and cyclic nucleotide phosphodiesterase;^41^ it also has been shown to inhibit chloride ion transport in red blood cells^42^ and organic cation transport 2 (OCT2) in the kidney.^43^ Halcinonide is a topical glucocorticoid that interacts with intracellular receptors to activate anti-inflammatory pathways as well as the activation of the G-protein–coupled Smoothened receptor^44^ in the plasma membrane.

All four drugs are small molecules (446–505 molecular weight) but only gefitinib and sorafenib are brain permeant and have clinical exposures in a concentration range (C_max_ 5–21 μM)^37,38^ similar to the effective concentration–response relationship in the α7 receptor (*Fig. 7*). In contrast, halcinonide is used topically and would not be expected to have significant systemic exposure and dipyridamide has C_max_ in the nanomolar range.^45^ Therefore, only gefitinib and sorafenib might be expected to show nicotinic receptor interactions clinically. It is interesting that gefitinib and sorafenib bear structural similarities to well-known α7 modulators. In particular, sorafenib, like the α7 modulators PNU-120596 and NS1738, is a urea compound with a halogenated aromatic ring. Gefitinib also bears a halogenated aromatic group, but does not have a urea backbone. In our assays, gefitinib, sorafenib, and NS1738 all modulate the receptor with similar potencies (EC_50_ values are in the range 1–10 μM, *Figs. 5* and *7*).

In summary, our results demonstrate the usefulness of an automated patch-clamp approach for identifying subtype-selective nicotinic receptor modulators within a well-characterized compound library. The pilot screen results provide evidence that the automated patch-clamp approach is capable of identifying compounds with a known nicotine receptor subtype activity. This screening approach may prove useful for identifying tobacco product constituents with nicotinic receptor subtype-selective activity and informing tobacco product regulatory science.

## Abbreviations Used

ACh: acetylcholine
AUC: area under the curve
CHO: Chinese hamster ovary
F-12: Nutrient Mixture F-12
DHβE: dihydro-β-erythroidine
DMSO: dimethyl sulfoxide
e-HTS: electrophysiology-based high-throughput screen
FDA: US Food and Drug Administration
FLIPR: Fluorometric Imaging Plate Reader
HBPS: HEPES-buffered physiological saline
HBSS: Hank's Balanced Salt Solution
hERG: human *Ether-à-go-go* related gene
IWB: IonWorks Barracuda
MLA: methyllycaconitine
OCT2: organic cation transport
PAM: positive allosteric modulator
PPC: population patch clamp
SD: standard deviation
SEM: standard error of the mean
